# The Contribution of Tau, Amyloid-Beta and Alpha-Synuclein Pathology to Dementia in Lewy Body Disorders

**DOI:** 10.4172/2161-0460.1000444

**Published:** 2018-08-10

**Authors:** David J. Irwin, Howard I. Hurtig

**Affiliations:** University of Pennsylvania Perelman School of Medicine, Department of Neurology Philadelphia PA, USA

**Keywords:** Lewy body disorders, Parkinson disease, Alpha-synuclein, Amyloid plaques, Tau neurofibrillary tangles, Dementia

## Abstract

Parkinson’s Disease (PD) and the closely related Dementia with Lewy Bodies (DLB) are due to the accumulation of pathogenic alpha-synuclein protein in brain cells manifest by heterogeneous motor and non-motor symptoms, including cognitive impairment and dementia. The majority of patients with Parkinson’s Disease develop Dementia (PDD) in late stages of the disease and have widespread neocortical distribution of alpha-synuclein pathology at autopsy, compared with PD without dementia, in which neocortical synuclein pathology is less prevalent. These three entities PD, DLB and PDD comprise a clinical spectrum, collectively known as Lewy Body Disorders (LBD). Recent investigations into the neuropathological basis of LBD have demonstrated that while synuclein pathology is the defining feature of these disorders, it is often accompanied by other age-related neurodegenerative pathologies. In particular, amyloid plaque and tau tangle pathology characteristic of Alzheimer’s Disease (AD) (~50% of all LBD patients have sufficient pathology at autopsy for a secondary neuropathologic diagnosis of AD), appear to contribute to cognitive impairment in LBD, and the combination is associated with a shorter interval between onset of motor symptoms and development of dementia and a shorter life span. Further, the co-occurrence of neocortical alpha-synuclein, tau and amyloid pathologies found at end-stage disease suggests a potential synergistic interaction of these individual pathologies in humans during life, mirroring experimental observations in animal and cell model systems that show how pathogenic species of synuclein fibrils can promote trans-synaptic spread of both tauopathy and synucleinopathy with strain-like properties. Newer post-mortem studies using digital methods to measure pathologic burden have highlighted distinct neocortical patterns of areas with relative higher density of tau pathology in LBD compared to AD that support these model data. The emerging field of cerebrospinal fluid and molecular imaging biomarkers of synuclein, amyloid and tau pathologies in LBD is contributing to a greater understanding of how the different pathologies evolve and interact to produce clinical heterogeneity in LBD. Future work to elucidate biologically meaningful clinical subgroups of synucleinopathy and its co-pathology must focus on the full clinicopathological spectrum of LBD and use validated biomarkers, when available, to design clinical trials based on the precise selection of homogeneous patient subgroups to maximize statistical power for detecting the impact of treatment.

## The Clinicopathological Spectrum of LBD and Overlap with AD

Parkinson’s Disease (PD) is a complex, progressive clinicopathological entity, clinically characterized by the variable presence of core extra-pyramidal symptoms that include bradykinesia, tremor, postural instability and rigidity [1]. Non-motor features of the disease are increasingly recognized and include autonomic failure, constipation, cognitive impairment, ansomia, Rapid eye-movement sleep Behavior Disorder (RBD) the latter two features usually occurring during the preclinical or prodromal stage of disease evolution as biomarkers and predictors of later onset of clinical symptoms [2]. Cognitive impairment and dementia in PD are highly common complications of late stage disease [3,4] and correspond to poor prognosis [5] and more profound patient disability [6]. The natural history of PD is notable for its marked variability among patients in the mixture of symptoms and the rate of progression from estimated time of onset to death (average duration of illness 15 years).

**Figure 1:**
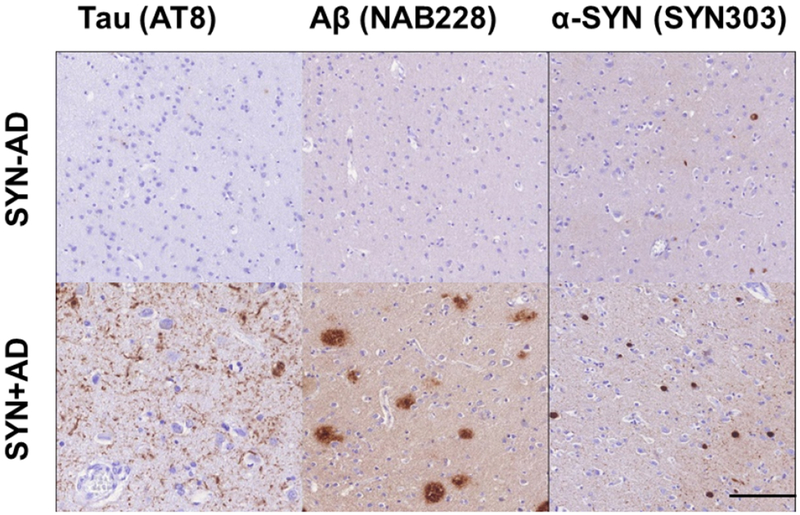
Mixed pathology in LBD. Photomicrographs depict neocortical pathology in LBD with “pure LBD” (TOP; SYN−AD) compared to LBD with AD co-pathology (BOTTOM; SYN+AD) with higher overall alpha-synuclein Lewy bodies and Lewy neurites in patients with SYN+AD. Scale bar= 100 μm.

**Figure 2:**
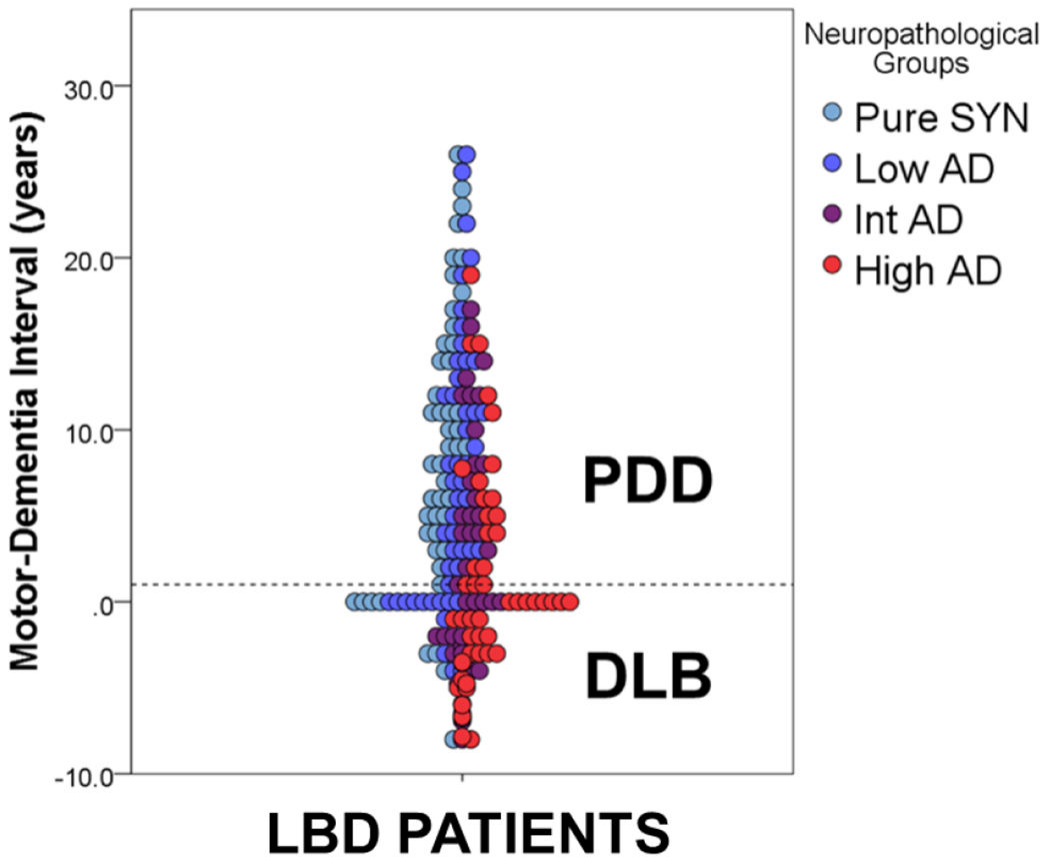
Clinicopathologic Spectrum of LBD. Scatterplot depicts individual patient data from a large LBD autopsy series (reproduced from Irwin et al, The Lancet Neurology 2017) showing the time interval from onset of motor symptoms to dementia in years defining the distinction of PDD and DLB (i.e. 1-year rule dashed line) compared to level of AD neuropathology found at autopsy. The arbitrary boundary between PDD and DLB does not differentiate patients with or without AD co-pathology. This clinicopathologic spectrum of synucleinopathy includes varying amounts of AD co-pathology which may influence clinical phenomenology as well as the response to future, more effectively targeted therapies.

The pathological hallmark of PD is the presence of pathologically misfolded alpha-synuclein (SYN) protein in the form of Lewy Bodies (LBs) and Lewy Neurites (LNs) in neurons of the Central Nervous System (CNS) (Figure 1). Modern immunohistochemical staining of SYN pathology has shown SYN pathology, not only in PD patients but also in the cortex of a large number of patients with clinically diagnosed AD at autopsy [7]. Historically several terms have been used to describe these mixed post-mortem findings, including the “Lewy body variant of AD” [8], but current nomenclature has become more refined. Indeed, the first consensus meeting to define the clinical syndrome of Dementia with Lewy Bodies (DLB) to predict the presence of neocortical SYN pathology in dementia patients occurred in 1995 [9] and has been further revised [10,11] to improve ante-mortem diagnostic accuracy. DTB is characterized by bradykinesia and other parkinsonian features, well-formed visual hallucinations, periodic fluctuations in cognition and RBD. These clinical features are often shared with PDD, with the distinction that the cognitive impairment in DTB starts within a year of the onset of motor symptoms-the arbitrary “one-year rule” as delineated by the third consensus on the definition of DTB [10]. This clinical distinction of PDD and DTB is currently a matter of debate, [1,12] because there is no pathologic substrate found at autopsy that can reliably differentiate these clinically defined disorders [13,14] (Figure 2). Moreover, both PDD and DTB share genetic risk [15–18] and preclinical/prodromal features (e.g. anosmia, RBD) [19–21]. However, the DLB clinical syndrome is currently considered a distinct entity by many experts because it is useful for prognosis in patient care and in educating the lay public that it has features that differentiate it from clinical AD.

Co-occurring AD associated amyloid-beta (Aβ) plaques and tau Neurofibrillary Tangles (NFTs) occur in 30-40% of patients with PD (Figure 1) and may contribute to clinical features of disease [22,23] Particularly Development of Dementia (PDD). Interestingly, PDD patients with greater levels of AD co-pathology may have a clinical phenotype that in some respects is more similar to DLB, with shorter time to dementia [13,22–24] and less prominent rest-tremor [25,26].

Thus, PD/PDD and DLB appear to exist on a clinicopathological spectrum of SYN pathology with varying degrees of AD co-pathology; hence the collective and more inclusive term Lewy body disorders (LBD) [8].

Attempts to cluster or sub-type PD based on clinical symptoms [27] may be helpful for prognosis but the underlying biological contributions to clinical heterogeneity of PD are currently unclear. Few studies include the full spectrum of LBD, since this often requires coordination of multimodal data collection across movement disorders and cognitive centers, where PD and DLB patients are often evaluated, respectively [22]. The current lack of a reliable method to detect and track SYN pathology *in vivo* makes detailed post-mortem clinicopathological work critical to elucidate the pathological substrates that contribute to cognitive impairment and clinical heterogeneity in LBD Here we review the recent literature on clinicopathological associations of dementia in LBD and include studies that have used *in vivo* biomarkers of AD pathology. The parkinsonian syndrome of multiple system atrophy, a related synculeinopathy, has a different neuropathological substrate of alpha-synuclein pathology primarily in oligodendrocytes [28] and is beyond the scope of this review.

## Alpha-Synuclein in Disease Pathogenesis of LBD

In 1997 two near-simultaneous reports found pathogenic mutations in the alpha synuclein (SNCA) gene encoding SYN protein in patients with hereditary PD [29] and pathogenic SYN protein as the primary constituent of LB/LNs in both PD and DLB [30]. These two important studies demonstrated that abnormal aggregation of SYN protein in the CNS is central to the disease process in PD and DLB (i.e. LBD). Landmark work by Braak and colleagues [31] defined a non-random distribution of SYN pathology in a large cohort of cross-sectional autopsied patients with clinically defined PD 1 (but not DLB) to develop a staging system of hypothesized disease spread within an individual. This model of spread of pathology from caudal brainstem regions in the medulla and the dopaminergic neurons of the substantia nigra of the midbrain to rostral subcortical and cortical regions largely maps to the clinical progression of disease in most patients from earliest symptoms to late stage dementia. Some deviations from this staging system have been described [32–35], including reports of the presence of post-mortem SYN pathology in some patients who died without evidence of clinical dementia or parkinsonism [36], which could suggest SYN pathology does not always contribute to neurodegeneration. Most of these patients with asymptomatic SYN pathology at autopsy (i.e. Incidental Lewy Body Disease or ILBD) have mild deposits in lower brainstem regions and are thought to be in a prodromal or preclinical stage of PD [37]. More direct evidence for pathogenicity of SYN pathology has been described recently in animal [38,39] and cell [40–42] model systems that have shown that pathogenic “seeds” of misfolded SYN pathology may induce native SYN misfolding and a cell to cell, prion-like propagation of SYN pathology between neurons. Strikingly, intracerebral injections of recombinant SYN protein alone can cause a motor phenotype and reduced survival in both transgenic [39] and “wild-type” [38] murine models associated with a time- and dose-dependent accumulation of SYN pathology in anatomically connected regions of the brain to recapitulate human disease. These findings of trails-neuronal spread of SYN pathology are reminiscent of the histopathological staging model of PD by Braak and colleagues [31] and have been replicated in animal models [43–45]. Further, several PD patients who received experimental fetal tissue grafts into the basal ganglia were found to have low levels of SYN pathology in grafted tissue at autopsy several years after implantation [46]. It is unclear if these aggregations of SYN pathology are spread from host PD brain tissue or developed independently due to other disease-related factors.

DLB staging is thought to follow a pattern of caudal-rostral spread of SYN pathology similar to the stages Braak described in PD from the brainstem to limbic and neocortical areas in most patients [11]. However, the findings of pathologic criteria for LBD at post mortem in some patients with dementia but no clinical parkinsonism [13] or striatal dopaminergic deficit on *in vivo* dopamine transporter imaging [47], could suggest that SYN pathology may have a different epicenter and pattern of spread during life in some DLB patients. Future work with more quantitative approaches to pathology staging and eventual *in vivo* markers of SYN pathology may further resolve these discrepancies.

Despite the tantalizing evidence of a prion-like mechanism of disease in PD and DLB, there is a sharp distinction between prion disease (i.e. caused by a proteinaceous infectious particle) [48] and SYN pathology, as there is currently no evidence that SYN pathology is infectious or can transmit between humans or animals, even in the setting of cadaver-derived human growth hormone recipients from pituitary samples which likely often contained SYN pathology as opposed to the rare contamination of prion protein which caused an epidemic of Cruetzfield-Jacob disease [49].

Observations of PD-like clinical symptoms and SYN pathology in adult patients with the multisystem lysosomal storage disease Gaucher’s disease with homozygous mutations in the Glucocerebrosidase A1 (GBA1) gene, led to the discovery of increased frequency of heterozygous GBA1 mutation carriers in LBD patients compared to the general population [50]. GBA1 mutations are associated with more rapidly progressive clinical parkinsonism [15,16], earlier onset of cognitive impairment [51,52] and advanced SYN pathology in the absence of AD co-pathology [13,17,53]. These findings have led to the consideration that autophagy (a lysosomal mechanism for disposing of altered proteins) and other mechanisms of protein homeostasis contribute to disease pathogenesis and an imperative to focus on GBA1 mutation carriers as an important biological subgroup of LBD.

Ongoing work to elucidate mechanisms of SYN pathology propagation and other potential downstream mechanisms of disease such as oxidative stress, synaptic dysfunction, disruption of axonal transport and inhibition of protein degradation pathways are vital to the development of disease-modifying therapies. Equally important for the success of clinical trials for these potential therapies is the ability to reliably detect biologically meaningful subgroups of LBD patients for homogenous patient recruitment when testing experimental therapies.

## Clinicopathological Correlates of Dementia in PD (PDD)

Cognitive impairment and dementia are common in PD and will develop in the majority of patients usually late in the course of the disease [3,4] but with significant heterogeneity in the timing of onset [13,22,23] and the rate of progression. Roughly [24] percent of PD patients are found to have mild-cognitive impairment at diagnosis [54] and are at high risk of developing later incipient dementia [55].

The pathologic substrates linked to dementia in PD are diverse and include a range of histopathological findings, including the distribution of Lewy SYN pathology in the neocortex [56–58], subcortical cholinergic loss [14,59], Cerebrovascular Disease (CVD) [60], Argyrophilic Grain Disease (AGD) [61], TAR DNA-binding protein 43 (TDP-43) inclusions in limbic structures [62] and hippocampal sclerosis of aging (HpScl) [63]. With standardized neuropathological assessments for AD and related disorders [64,65] there is increasing recognition of mixed or multiple pathologies in LBD and other neurodegenerative diseases, along with the non-demented aging spectrum.

Large autopsy-series of PD find that the distribution of SYN pathology in a neocortical pattern is a strong correlate of dementia during life [23,56–58,66]. We previously performed a deep pathological phenotyping of a large cohort of PD patients (n=140), who died with or without dementia and included systematic evaluation of SYN, Tau NFTS, Aβ plaque, TDP-43, CVD and HpScl, as well as genotyping for Apolipoprotein E (APOE) and MAPT tau haplotype [23]. We found that each of these pathologies was associated with dementia independently. However, using a multivariate approach, we found the strongest correlate with dementia was the neocortical burden of SYN pathology, suggesting that propagation of caudal to rostral spread of SYN pathology [31] as proposed by Braak, is a main driver of the emergence of dementia in PD. Thus, patients with PDD at end-stage disease have indistinguishable pattern of wide-spread SYN pathology compared to DLB. We also found an association of the APOE £4 genotype with dementia that was independent of Aβ plaque and tau NFT pathology. This suggests that the APOE genotype may confer risk for SYN pathology in a manner distinct from AD pathology. APOE £4 has been found in a greater frequency in both patients with “pure” (no co-pathology) LBD and those with LBD and AD co-pathology compared to the general population [18], and some post-mortem studies find an independent association of APOE £4 with SYN pathology [58,67], which further reinforces a link between this common risk variant and LBD.

## AD Co-Pathology across the Spectrum of LBD

Several other large-scale studies of autopsied PD brains have found a strong influence of co-existent AD pathology on cognitive status [4,23,24,60,66,68–70]. One study found that the combination of SYN with Aβ plaque and tau NFT pathology is the strongest correlate with dementia in PD [66], and another, using ante-mortem neuropsychological data to define cognitive status in PD70, similarly found a combination of SYN and AD co-pathology to be the most influential. We and others find that high levels of AD co-pathology in PD are nearly universally associated with PDD (i.e.<10% of PD without dementia has significant co-morbid AD at autopsy23) and also are associated with higher cortical SYN compared to PD patients without significant AD co-pathology [13,23,57,58,60,70,71]. Moreover, patients with PDD who have significant AD co-pathology tend to be older, have a shorter time interval to develop dementia after onset of parkinsonian motor symptoms and a shorter life span [23,68].

The Sydney longitudinal, multicenter study of PD followed a large cohort of patients with PD prospectively and found a similar subgroup of older PD patients with more aggressive disease [4,24,69]. These data suggest that age-related factors, including cerebrovascular disease and AD co-pathology, may increase the risk of earlier onset of dementia in the course of disease that more closely approximates DLB on the spectrum of LBD *vs* PD (Figure 2). Indeed, the majority of DLB patients (>70%) in a large, multi-center cohort were found to have a medium to high-level of AD neuropathologic change at autopsy [13]. A minority of those patients with DLB who had a pattern of “pure” SYN pathology (i.e. no Alzheimer co-pathology) were carriers of the GBA1 mutation or had other co-pathologies, including CVD, indicating that while AD co-pathology influences the DLB phenotype, it does not do so in every case.

In our study of patients with LBD spectrum disease (PDD/DLB), we found that tau NFT pathology is the strongest correlate of reduced survival and earlier time to dementia [13], while others find AB plaque or SYN pathology to be a strong correlate for the timing of dementia58 and survival [72]. Since all three pathologies (i.e. Tau, Aβ and SYN) are correlated in the neocortex in LBD [13,25], sample size and varying methodologies used to detect and quantify these pathologies could contribute to discrepancies in outcome. These limitations notwithstanding, AD co-pathology and associated neocortical spread of SYN appear to confer an overall worse prognosis in LBD.

Few data exists, relating post-mortem pathology to specific ante-mortem clinical features or cognitive profiles. Cognitive and motor features of LBD are heterogenous, [10,73,74] and there is currently no clear clinical phenotype to distinguish AD co-pathology in LBD. Data-driven clusters of PD patients have shown that a clinical subgroup with less prominent rest tremor and more prominent postural instability is linked to higher Aβ and SYN pathology in the neocortex [26] and to clinical biomarkers of AD [27]. Few autopsy-confirmed studies with ante-mortem quantitative neuropsychological testing data exist to test the cognitive domains that are impaired in LBD with and without AD co-pathology [25,75–77]. Some studies suggest that temporal-lobe mediated naming tasks may be worse in LBD with mixed AD co-pathology compared to pure LBD [25,76,77]. We find increasing tau pathology is a strong correlate of worsening cognitive scores in LBD patients with dementia, [25] and others find that a combination of pathology in the prefrontal cortex and temporal lobe is a strong correlate of cognitive decline [70].

Using digital methods to measure the burden of pathology parametrically, we have found similar levels of Aβ pathology but much lower levels of tau pathology in LBD with AD co-pathology than seen in autopsy-confirmed clinical AD, but the tau pathology has greater concentration in the temporal lobe [25]. Further, we have also found that overall SYN pathology in LBD with AD co-pathology is highest in the frontal and temporal lobes. This novel digital investigative methodology suggests that tau may accumulate in a manner that is distinct from AD in LBD and share a locus of pathology with SYN in the temporal lobe.

It is impossible to deduce the timing or mechanism for these observations from human post-mortem histology alone, but several strands of evidence suggest a link between tau and SYN pathology in LBD. First, the Contorsi kindred of autosomal dominant PD patients with the Ala53Thr pathogenic mutation in the SNCA gene was found to have high levels of tau pathology in addition to SYN [78]. Genetic variation in the H1 haplotype of the tau gene MAPT has been linked to increased risk for PD [79] and DLB [80], as well as the accumulation of cortical SYN pathology [81] and the risk of dementia in PD in some studies [82–84], but not others [23]. An *in vitro* cell model [85,86] and transgenic SYN murine models [86] suggest that tau and SYN pathology can accelerate co-polymerization of tau. More recently, novel experiments in a cell model have demonstrated two distinct strains of recombinant SYN fibril preparations, including one strain that can induce both tau and SYN pathology [42]. Moreover, the use of specific novel monoclonal antibodies to study these distinct strains of pathogenic SYN has detected unique patterns of pathology in human LBD samples [87]. This growing body of work provides compelling evidence to suggest synergy between tau and SYN pathology.

Some evidence suggests that increased AD co-pathology in LBD may mask the usual cognitive features in DLB of visual hallucinations and cognitive fluctuations [88,89]. Indeed, the clinical criteria for DLB are specific but less sensitive to detect neocortical SYN Lewy pathology at autopsy [90]. It is likely that the majority of patients with an AD clinical amnestic syndrome that have widespread neocortical SYN pathology at autopsy may represent a “limbic predominant” pattern originating from the amygdala [35], as brains from these patients have less subcortical and brainstem SYN pathology and extracranial SYN pathology in the peripheral nervous system [71] typical of LBD [91]. Roughly 50% of patients with sporadic and hereditary AD have SYN co-pathology in the amygdala and other limbic regions at autopsy [7,92]. Further, brains from patients with clinical AD and co-existent SYN pathology have higher hippocampal tau than LBD [93], suggesting these patients are biologically distinct from clinical LBD (i.e. PD, PDD, DLB). It is also possible that a subset of clinical AD patients with neocortical SYN pathology reported at autopsy could be misdiagnosed during life, since clinical diagnostic accuracy for DLB based on the one year rule is currently less than optimal [94]. Thus, the pathological spectrum of SYN pathology also includes a large proportion of AD patients, making clinical distinction of AD patients with and without SYN pathology challenging. Recent revised clinical criteria for DLB10 await validation and might improve the ante-mortem detection of SYN pathology in dementia of any type.

## AD Biomarker Studies in LBD

*In vivo* biomarkers to detect signatures of Aβ plaque and tau tangle pathology in AD have been studied in LBD and provide converging evidence to the post-mortem data discussed above. Assays for pre-mortem cerebrospinal fluid (CSF) measurements of total-and phosphorylated forms of tau (t-tau, p-tau) and Aβ1-42 show direct associations with post-mortem tau NFT and Aβ plaque pathology in AD [95] where low Aβ1-42 and high t-tau and p-tau represent a signature of AD pathology. This pattern of CSF analytes can robustly differentiate between AD from non-demented controls [96] and predicts clinical progression to a diagnosis of AD in patients with Mild Cognitive Impairment (MCI) [97]. In PD, one prospective study found that low levels of CSF Aβ1-42 predict cognitive decline, [98] and cross-sectional studies have found that AD biomarkers in CSF are associated with cognitive impairment [99–102]. Similarly, in DLB, biomarkers for AD in CSF show association with poor prognostic clinical markers such as falls, institutionalization and shortened life span [103]. Across the LBD spectrum, the CSF biomarker signature of AD is found increasingly more common between groups of PD, PDD and DLB (reviewed in 22), which resemble frequencies of AD co-pathology seen in large autopsy studies (i.e. <10% PD, 40% PDD, >70% DLB) [13,22,23]. In early clinical PD, levels of t-tau and p-tau in CSF are lower than in control patients and are highly correlated with CSF measurements of total-alpha-synuclein [104], further suggesting that the accumulation of tau pathology and pathogenic species released into CSF may be distinct in LBD compared to AD and normal aging. Despite these differences in low t-tau/p-tau levels in early PD, cross-sectional samples of more advanced PD/PDD and DLB have found wide ranges of CSF AD biomarker values, with some overlap of individual data points with both controls and AD patients [105]. There is little autopsy confirmed data on the validity of AD CSF biomarkers in LBD. These few studies that do exist suggest that AD co-pathology may influence CSF biomarker level associated with autopsy proven AD [106]. We found a direct association with post-mortem measurement of CSF t-tau and Aβ with post-mortem severity of Aβ and tau pathology, as well as correlations with Aβ and the t-tau/Aβ ratio with SYN pathology [107]; further suggesting synergy between AD and SYN co-pathology. Preliminary data from this study suggest that clinical diagnostic accuracy to distinguish LBD with AD co-pathology from “pure” LBD ante-mortem using a cut-point of the t-tau/Aβ ratio may be in a range suitable for clinical trials (>80% sensitivity/specificity) [107]. Differentiating clinical AD with and without SYN co-pathology using CSF biomarkers is more challenging; however, some studies suggest CSF alpha-synuclein levels may improve diagnostic accuracy [105,108]. CSF alpha-synuclein assays are not yet fully reliable because of the risk that leaked blood during lumbar puncture can contaminate CSF measurements, requiring the need to account for hemoglobin levels in CSF [109]. Further, there is a large overlap of SYN levels between control and groups of PD patients with PD, making interpretation of the values for individual patients difficult [110]. Newer assays for phosphorylated 108 or oligomeric [111,112] forms of CSF synuclein are in development and may be more sensitive to disease-specific forms of alpha-synuclein. Finally, new approaches using patient CSF samples to seed and induce pathological misfolding of recombinant or native synuclein in a similar manner to prion disease testing (i.e. real-time quaking inversion; RT-QuIC assays) [113,114] are promising for a specific marker of SYN pathology *in vivo.* Future studies to replicate and validate these assays must be done before an authentic SYN-specific marker can be trusted for utility as a precise tool for selecting accurately diagnosed patients for clinical trials of more effective therapies in LBD

Neuroimaging is another technique for studying AD co-pathology in LBD. Hippocampal atrophy on structural MRI has been linked to tau pathology in AD [115] and predicts cognitive impairment [116] and reduced survival in DLB [117]. Positron Emission Tomography (PET) using tracers specific to Aβ plaque pathology finds a similar frequency of amyloid pathology in PDD and DLB [118] but less abundantly in PD without dementia, in keeping with post-mortem studies described above [13,22,23]. Further, PET amyloid tracer binding appears to relate to post-mortem AB neuritic plaque burden in PD [119]. Moreover, PET imaging to detect tau pathology is emerging and flortaucipir, a novel PET tracer directed at AD tau pathology has been examined in several studies of LBD. These studies have generated data to suggest that tau pathology is overall lower in LBD than in AD, but in a distribution distinct from the typical localization in AD in posterior temporoparietal [120,121] and primary mot or/sensory cortices [122]. Further, PET tau binding correlates in general with cognitive impairments across the LBD spectrum of PD, PDD and DLB [121] but in focused studies of patients with early PD and MCI tau binding is negligible in those with the least amount of altered cognition [123,124]. Moreover, PET tau binding was found only in those patients with PET amyloid positivity [122,123], which conforms with post-mortem work showing advanced tau pathology in LBD largely in the setting of high-level Aβ pathology [22]. A small subset of patients with LBD evaluated by PET using a combination of amyloid and tau tracers has shown tau pathology but not amyloid plaque formation [120,121]. This discrepancy may be due to the insensitivity of the PET amyloid tracer to milder, more diffuse plaque pathology; or it could represent further evidence that tau pathology alone is a distinct form of co-pathology accruing in LBD. Additional studies of AD pathology in LBD with emerging biomarkers in prospective cohorts followed to autopsy is likely to validate these ante-mortem imaging results. Finally, there is an urgent need for a reliable way to detect and track SYN pathology during life to fully solve the timing and progression of SYN-associated neurodegeneration in both “pure” LBD and LBD with AD co-pathology.

## Conclusion

The clinical components of the LBD spectrum make up a complex clinicopathological entity with diverse cognitive and motor features (Figure 2) and widespread distribution in the CNS of SYN pathology, often accompanied by AD co-pathology. When AD co-pathology is significant, it likely contributes to the clinical phenotype in ways not yet fully understood, including the timing of dementia and overall survival. Tau pathology, in particular is a strong correlate of cognitive impairment and survival and may be induced by the propagation of pathogenic strains of SYN pathology. While there is debate over the clinical distinction of PD and DLB, a more salient issue may be the ante-mortem detection and differentiation of LBD patients with AD co-pathology from those with “pure” SYN pathology. LBD patients with AD co-pathology and worse prognosis may influence clinical trial outcomes for both symptomatic therapies as well as emerging SYN-targeted disease-modifying therapies; thus, it is pertinent for clinical trial designs in LBD to consider stratification of enrollment based on AD and SYN biomarker profiles. In the final analysis, significant advances in meaningful therapies will depend on a more precise understanding of how the diverse spectrum of molecular pathologies in LBD interact to produce clinical neurodegeneration.
